# Toward a theory-led meta-framework for implementing health system resilience analysis studies: a systematic review and critical interpretive synthesis

**DOI:** 10.1186/s12889-022-12496-3

**Published:** 2022-02-12

**Authors:** Zeynab Foroughi, Parvin Ebrahimi, Aidin Aryankhesal, Mohammadreza Maleki, Shahram Yazdani

**Affiliations:** 1 School of Health Management and Information Sciences, Iran University of Medical Sciences, Tehran, Iran; 2 Virtual School of Medical Education and Management, Shahid Beheshti University of Medical Sciences, Tehran, Iran

**Keywords:** Health system, Resilience, Meta-framework, Critical interpretive synthesis

## Abstract

**Introduction:**

The variety of frameworks and models to describe resilience in the health system has led researchers and policymakers to confusion and the inability to its operationalization. Therefore, the purpose of this study was to create a meta-framework using the Critical Interpretive Synthesis method.

**Method:**

For this purpose, studies that provide theories, models, or frameworks for organizational or health system resilience in humanitarian or organizational crises were systematically reviewed. The search strategy was conducted in PubMed, Web of Science, Embase, and Scopus databases. MMAT quality appraisal tool was applied. Data were analysed using MAXQDA 10 and the Meta-ethnography method.

**Results:**

After screening based on eligibility criteria, 43 studies were reviewed. Data analysis led to the identification of five main themes which constitute different framework dimensions. Health system resilience phases, attributes, tools, and strategies besides health system building blocks and goals are various dimensions that provide a systematic framework for health system resilience analysis.

**Discussion:**

This study provides a systemic, comprehensive framework for health system resilience analysis. This meta-framework makes it possible to detect the completeness of resilience phases. It examines the system’s resilience by its achievements in intermediate objectives (resilience system attributes) and health system goals. Finally, it provides policy solutions to achieve health system resilience using tools in the form of absorptive, adaptive, and transformative strategies.

**Supplementary Information:**

The online version contains supplementary material available at 10.1186/s12889-022-12496-3.

## Introduction

Health system resilience is known as a way to achieve universal health coverage (UHC) through health system strengthening against chronic challenges and acute shocks [1]. Although the term “resilience” has been employed in engineering, psychology, and ecology sciences for more than a decade, it has been used in health system research in recent years [2]. The negative consequences of the Ebola virus outbreak on the health system in West Africa, including interruption in the delivery of essential health services and losses of many lives, contributed to the popularity of the health systems resilience concept [3, 4]. In the same way, the COVID-19 pandemic has increasingly raised the clarity of the need for resilience in health systems [5]. By definition, health system resilience is the ability of the system to prepare for and respond to sudden shocks and everyday challenges and its capacity to absorb deteriorations, adapt, and transform the health system to cope with them [5, 6].

The results of a concept analysis suggested that there is fragility in applying the health system resilience concept. Different researchers use various frameworks for analysing health system resilience [2]. Hence, there is no specific suggestion about achieving a resilient health system [7]. For example, Hollnagel focused on the concept of resilience engineering and defined four resilient health system capabilities as anticipating, monitoring, responding, and learning [8]. Kruk et al. also introduced the attributes of health system resilience, considering it as living organisms, including aware, diverse, self-regulating, integrated, and adaptive [9]. Blanchet et al. presented health system resilience capacities, including absorptive, adaptive, and transformative capacities [10].

The lack of a unified framework for studying health system resilience can hinder its operationalization [11]. Therefore, integration and unification of the current health system resilience frameworks are necessary for realizing its potentials [12]. Hence, we conducted this study to achieve a comprehensive framework for analysing health system resilience. This study applied reviewing and synthesis of the existing conceptual frameworks and finding the common elements among them. The initial study question was about detecting different components of a resilient health system that should be considered during resilience system analysis studies.

## Materials and methods

This systematic review was conducted and is reported according to the Preferred Reporting Items for Systematic Reviews and Meta-Analyses (PRISMA) guidelines (Supplementary File 1).

### Eligibility criteria

The inclusion criteria were all studies published in English with any quantitative or qualitative design and book chapters. Included studies should be introduced or defined a resilience theory, model, or framework in organizational or health system context and/or in connection with a humanitarian or organizational crisis. Also, reports published by international organizations were included. Studies related to ecological, or psychological resilience were excluded. Further, empirical studies that did not mention resilience system components were excluded.

### Information sources & search strategy

We applied the search strategy in PubMed, Web of Science, Embase, and Scopus databases in November 2020. Keywords include Resilience, resilient combining with organiz*/organis* or system or “health system” and theory, framework, model, and synonyms. Also, reference checking of detected studies and hand searching of related journals were conducted. The complete search strategies for all databases are presented in Table 1.

**Table 1:** The search strategies for all databases and their results

Database	Search Strategy	Document Results
WOS	TITLE: (Resilience OR resilient) AND TITLE: (System OR model OR framework OR theory OR organiz* OR organis* OR concept) AND TOPIC: (healthcare OR “health care” OR “health system” OR “health sector”) Timespan: All years. Indexes: SCI-EXPANDED, SSCI, A&HCI, CPCI-S, CPCI-SSH, BKCI-S, BKCI-SSH, ESCI	230
PubMed	(Resilience[Title] OR resilient[Title]) AND (System[Title] OR model[Title] OR framework[Title] OR theory[Title] OR organization[Title] OR organizational[Title] OR concept[Title])	701
Embase	(resilient:ti OR resilience:ti) AND (framework:ab,ti OR model:ab,ti OR program:ab,ti OR indicator:ab,ti OR index:ab,ti) AND (system:ab,ti OR organization:ab,ti OR organizational:ab,ti OR theory:ab,ti)	834
Scopus	(TITLE (*resilience* OR *resilient*) AND TITLE (*system* OR *model* OR *framework* OR *theory* OR organiz* OR organis* OR *concept* OR *program* OR *indicator*) AND TITLE (*healthcare* OR *“health care”* OR *“health system”* OR *“health sector”*))	181

### Selection process

Duplicate documents were removed using Endnote software. Tow authors (ZF and AA) screened retrieved studies. In the first stage, screening was regarding the relevance of the title and abstract. Next, the studies’ full-texts were screened in terms of compliance with the inclusion and exclusion criteria. The authors discussed controversies in the screening process.

### Quality appraisal

Quality assessment was based on Mixed Methods Appraisal Tool (MMAT), VERSION2018. This tool is applicable for quality assessment of systematic mixed studies review that needs to quality assessment of various quantitative, qualitative, and mixed-method studies [13]. This tool rates studies with a five-point scale: 0, 25, 50, 75, and 100 (where 100 indicates the highest level of quality). Moreover, non-empirical studies that did not have an explicit research question based on the definition of this checklist and did not respond to the collected data were excluded from the appraisal process. Two researchers conducted the ratings. Disagreements were resolved using discussion and a third researcher. Given that the present systematic review was qualitative, no study was excluded for having a low rating. The results of study ratings are applied in the interpretation of data.

### Data collection and analysis

This study used the critical interpretive synthesis, as an empirical method with the aim of theorization. The method is specified for analyzing studies with diverse qualitative and quantitative methods [14]. The motivations for using this method were its qualitative principles [15], capability to analyze and interpret complex literature [16], and its ability to develop a new concept, framework, or model that is the aim of the present study [15, 17]. Critical interpretive synthesis deploys meta-ethnography strategies [18]. While the traditional reviews focus on aggregative synthesis, the focus of critical interpretive synthesis is on the interpretive synthesis [19]. Data analysis was based on the meta-ethnography method using MAXQDA 10 software. Applying this method in the first step, the key themes in each study were identified, then the authors translated the themes from various studies into each other. In the second step, controversies among models were detected and explained. Finally, a general and comprehensive interpretation was developed.

## Results

**Fig. 1:**
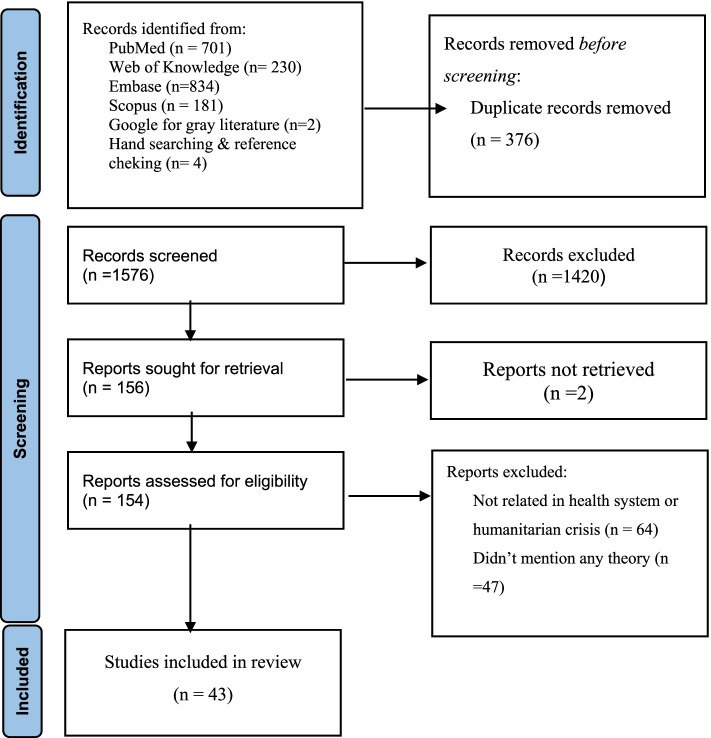
PRISMA 2020 follow diagram

The search strategy contributed to the identification of 1567 studies. After the two-stage screening, 43 studies met eligibility criteria. The PRISMA flow diagram of detected studies was presented in Fig. 1.

### Quality appraisal

Since the present study sought to identify existing theories, a large number of detected studies (35%) were non-empirical. Almost 53% of studies gained a high-quality score (100 or 75). Only 11% of studies were in low rating.

### Synthetic model

Review and critical interpretive analysis of studies indicated five main themes. These themes constitute the health system resilience analysis meta-framework. Accordingly, our synthetic model consists of health system resilience phases, attributes, main strategies, tools, and relationships with health system building blocks. Detected themes and related studies are presented in Table 2.

**Table 2:** Detected themes and related studies

	Quality appraisal	Phases					Attributes			
		Anticipation	Preparation	Response	Recovery	Growth	Awareness	Surge capacity	Flexibility	Resistance
(Meyer et al., 2020) [20]	100							*		
(de Carvalho et al., 2012) [21]	NA**									
(Thomas et al., 2013) [22]	25									
(Falegnami et al., 2018) [14]	100	*		*						
(Shirali et al., 2016) [23]	75		*				*		*	*
(Haghighi and Torabi, 2018) [24]	NA		*				*	*	*	
(Patriarca et al., 2018) [25]	100	*		*						
(Ho et al., 2016) [26]	NA		*					*		
(Therrien et al., 2017) [27]	NA			*			*	*	*	
(Barker et al., 2020) [28]	100						*	*	*	
(Anderson et al., 2020) [29]	25	*		*						
(Najarian and Lim, 2019) [30]	75	*		*	*			*	*	*
(Omidvar et al., 2017) [31]	50		*				*		*	
(Barasa et al., 2017) [32]	NA			*			*			
(Blanchet et al., 2017) [10]	NA	*					*	*		
(Biddle et al., 2020) [12]	100	*					*	*		
(Cristian, 2018) [33]	NA		*	*	*		*	*		
(Ybarra, 2019) [34]	NA			*					*	
(Fridell et al., 2020) [7]	75		*	*				*	*	
(Gilson et al., 2017) [35]	75			*			*			
(Frisbie and Converso, 2016) [36]	100						*	*		
(Moran, 2016) [37]	100			*			*	*		
(Witmer and Mellinger, 2016) [38]	100						*			
(Rangachari and L Woods, 2020) [39]	NA			*	*		*			
(Kong and Simonovic, 2019) [40]	75		*		*					*
(Khan et al., 2018) [41]	100								*	
(Argyroudis et al., 2020) [42]	75				*				*	*
(Clay-Williams and Braithwaite, 2019) [43]	NA	*		*						
(Kagwanja et al., 2020) [44]	100			*			*			
(Pęciłło, 2016) [45]	NA	*		*	*					*
(Vos et al., 2020) [46]	100	*		*					*	
(Rios et al., 2020) [47]	75						*	*	*	
(Dos Santos et al., 2020) [48]	100	*	*	*	*					*
(Fallah-Aliabadi et al., 2020) [49]	100		*	*	*					*
(Woods et al., 2014) [50]	NA	*		*						
(Specking et al., 2019) [51]	25				*				*	*
(Tumusiime et al., 2020) [52]	50		*	*	*	*	*			
(Wiig et al., 2020) [53]	NA					*		*	*	
(Nuzzo et al., 2019) [54]	100		*		*			*	*	
(Thomas et al., 2020) [5]	NA		*	*			*	*	*	
(Turenne et al., 2019) [2]	100			*	*				*	*
(Iflaifel et al., 2020) [55]	100	*		*			*		*	*
OECD, 2014 [56]	NA	*	*	*	*	*			*	

	Quality appraisal	Attributes		Tools					Main strategies		
		Access to resources	Collaboration and Coordination	Risk analysis	Planning	Monitoring	ICS*	Learning	Absorptive	Adaptive	Transformative
(Meyer et al., 2020) [20]	100		*				*				
(de Carvalho et al., 2012) [21]	NA**		*		*	*		*			
(Thomas et al., 2013) [22]	25								*	*	*
(Falegnami et al., 2018) [14]	100					*		*			
(Shirali et al., 2016) [23]	75					*		*			
(Haghighi and Torabi, 2018) [24]	NA		*					*	*		
(Patriarca et al., 2018) [25]	100					*		*			
(Ho et al., 2016) [26]	NA		*			*		*			
(Therrien et al., 2017) [27]	NA	*	*					*			
(Barker et al., 2020) [28]	100		*				*			*	
(Anderson et al., 2020) [29]	25					*	*	*		*	
(Najarian and Lim, 2019) [30]	75								*	*	
(Omidvar et al., 2017) [31]	50							*			
(Barasa et al., 2017) [32]	NA	*							*	*	*
(Blanchet et al., 2017) [10]	NA		*				*		*	*	*
(Biddle et al., 2020) [12]	100		*						*	*	*
(Cristian, 2018) [33]	NA		*				*	*			
(Ybarra, 2019) [34]	NA							*		*	
(Fridell et al., 2020) [7]	75	*	*			*	*				
(Gilson et al., 2017) [35]	75	*							*	*	*
(Frisbie and Converso, 2016) [36]	100	*	*					*			
(Moran, 2016) [37]	100	*	*								
(Witmer and Mellinger, 2016) [38]	100		*						*		*
(Rangachari and L Woods, 2020) [39]	NA										
(Kong and Simonovic, 2019) [40]	75										
(Khan et al., 2018) [41]	100	*	*	*	*	*	*	*			
(Argyroudis et al., 2020) [42]	75	*									
(Clay-Williams and Braithwaite, 2019) [43]	NA					*		*			
(Kagwanja et al., 2020) [44]	100	*							*	*	*
(Pęciłło, 2016) [45]	NA							*	*		
(Vos et al., 2020) [46]	100							*			
(Rios et al., 2020) [47]	75		*							*	
(Dos Santos et al., 2020) [48]	100	*			*					*	
(Fallah-Aliabadi et al., 2020) [49]	100	*	*		*		*	*			
(Woods et al., 2014) [50]	NA					*		*			
(Specking et al., 2019) [51]	25	*			*				*	*	
(Tumusiime et al., 2020) [52]	50		*					*			*
(Wiig et al., 2020) [53]	NA		*					*			
(Nuzzo et al., 2019) [54]	100	*	*		*		*				
(Thomas et al., 2020) [5]	NA	*	*			*	*	*	*	*	*
(Turenne et al., 2019) [2]	100										
(Iflaifel et al., 2020) [55]	100	*					*	*		*	
OECD, 2014 [56]	NA		*					*	*	*	*

*Information and Communication Systems

**Not Applicable

### Health system resilience phases

The included studies referred to five main interrelated phases in system resilience.

#### Phase I: anticipation

Anticipation is the first phase of any activity to achieve health system resilience [7, 30, 39, 53, 55, 57–59]. Health systems need to acknowledge their vulnerabilities before providing any response [48, 60]. In this phase, risk analysis and risk assessment methods will be applied [41]. This concept is referred to as forecasting, foresight, predicting, or detection of adverse events [30], potential system vulnerabilities [30, 61], uncertainties [10], deteriorations [53], or possible scenarios using various tools such as simulation methods [61]. This phase requires a powerful information system as well as good collaboration and coordination [41].

#### Phase II: preparation

In the preparation phase, based on the type of risk and system vulnerabilities identified, the essential capacities of the health system will be promoted [5, 6, 53]. During this phase, planning [32], different response plans [5], scenario exercises, leadership and command structure in emergencies, legal preparations, and control and monitoring structures are defined [54].

#### Phase III: response

Responding is defined as the appropriate reaction to changing circumstances and is essential to guarantee good performance [2, 7, 10, 30, 32, 46, 61, 62]. It requires the proper anticipation and preparations [45]. This phase should include all health system building blocks (Leadership & governance, service delivery, human resources, financing, drug & medical equipment, and information system) [10]. Moreover, the quality and the rapidity of decisions depend on suitable collaboration and coordination [10, 25].

#### Phase IV: recovery

The majority of studies referred to recovery as the last phase to achieve system resilience [30, 33, 39, 49, 54, 58]. Indeed, resilient systems have plans to return to stable states [30]. Self-regulation, another expression of this phase, refers system’s ability to reduce performance deteriorations and absorb environmental changes [28, 47, 52, 59]. Recovery level and recovery time are two evaluation indicators of this phase [61]. The activities of this phase have a long-term view and require policymaking, analysis, and evaluation skills to detect optimal applied response plans. The recovery phase improves system preparedness through reactive and proactive activities to adapt old structures or create new ones [57, 63].

#### Phase VI: growth

Some studies considered growth as the last phase of the creation of resilient systems [59]. In other words, system capabilities will improve during the challenging condition through learning tools and growth that occurs in the long term [57, 59, 64].

### Health system resilience attributes

#### Awareness

A resilient system with a good understanding of its existing situation, including the operational environment, needs, and resources, can well predict future internal and external changes as well as timely informing decision-makers and the public about crisis and its potential implications on the system [10, 20, 23, 31, 48, 65]. Kruk et al. named this attribute “cognitive capacity” and defined it as the ability of the system to detect and interpret shocks and chronic challenges (sense-making) [32]. Also, Blanchet et al. considered system “knowledge” and defined it as the capacity of collecting, analysing, and interpreting the information in addition to awareness [10]. Situation awareness requires appropriate collaboration and coordination between different stakeholders in the system [41]. Besides, a powerful information system with the ability to early detection, analyzing, and share information, including weak change signals from various internal and external parts of the health system, is a necessity for an aware health system, as a complex adaptive system [41, 52, 61]. In brief, awareness is composed of three main components of predicting, monitoring, and communicating, leading to sensemaking (ability to detect and interpret system changes) [28, 37, 47, 58, 59].

#### Surge capacity

The ability to increase the capacity of various system components (Health system six building blocks) in response to shocks or everyday challenges defines surge capacity [20, 25, 27, 54]. Barasa et al. specified this attribute as “behavioural capacity” which is the system’s ability to respond to unforeseen circumstances based on learnings and preparations [44]. Also, some studies mentioned the term “agility” and the ability to change in an uncertain environment [61, 64, 65]. Hence, a resilient system will adapt the capacities of the health system building blocks upon understanding weak signals through its learning tools and awareness attribute [20, 27]. Learning tools assist the system in predicting appropriate and adequate capacity in different situations [44].

#### Flexibility

A resilient health system has flexible managerial and executive structures and coping strategies which can be adjusted in an emergency [23, 28, 47, 54, 66]. In another definition, flexibility refers to the extent and rapidity of system adaptations to shocks without failure in system processes [61]. In this regard, studies referred to “Redundancy” which is the existence of various executive solutions, choices, and adaptive options under pressure conditions [42, 58, 65]. Also, redundancy is about continuity of services due to the presence of various staff and equipment that can perform specific tasks [61]. In addition, some researchers mentioned the “Diversity” of health system building blocks in different situations [28, 47, 52, 58]. Flexibility relates to the anticipation of uncertainties and is dependent on the learning tool and transformative strategies [67, 68]. Provision of safety inventory, multiple sources of supplies, and multipurpose equipment and staff are some examples of flexibility [68].

#### Resistance

This attribute is also known as “Robustness”, “stability”, or “coping” is about the system withstanding challenges and deteriorations [30, 42, 49, 61, 65, 66]. This attribute results from the recovery phase and means the ability to maintain the main system characteristics and continuity of its critical operations [59].

#### Access to resources

Resource availability or resourcefulness, including human and financial resources, materials, and technologies specified to face a crisis, is an empowering factor to achieve system resilience [42, 66]. The resilience system can mobilize, acquire and distribute essential resources and assets against the crisis. Also, it has appropriate resource allocation strategies that ensure timely access to resources [37, 48, 49, 61].

#### Collaboration and coordination

Several studies referred to collaboration and coordination as an important resilient system attribute [2, 10, 33, 41, 48]. Collaboration and coordination are among the properties of an integrated system against crisis and emergencies [41, 52, 65, 68]. Health system resiliency is not possible without a proper understanding of the role of its actors and stakeholders [28, 53]. Resiliency requires predetermined coordination mechanisms in which power dynamics are considered [2, 20, 49]. Collaboration and coordination influence preparation, response, and recovery phases and contribute to flexibility through the creation of a shared understanding of the situation [41, 67, 68]. It also will be influenced by information and communication system [20]. Blanchet et al. called this attribute “capacity to manage interdependency” which means managing stakeholders’ cross skills and feedbacks [10].

“Community engagement” constitutes a necessary aspect of collaboration and coordination in most resilience system theories and models [28, 33, 41, 54]. Community engagement and community involvement in decision makings can lead to cultural consideration in the implementation of policies and programs, creating a shared understanding with community and trust [28, 41, 54].

### Health system resilience tools

#### Risk analysis

Detecting system vulnerabilities and planning for preparation and mitigation of adverse effects requires risk analysis tools and measuring the magnitude and severity of risks [63]. Therefore, risk analysis is the first step toward awareness and requires good collaboration and coordination and promoting information and communication systems to anticipate them [41, 49].

#### Planning

The resilient health system should have plans to solve the aftermath problems of the crisis [48, 54, 63]. Adaptable responses and resource allocation plans are necessary to cope with rapidly changing circumstances and become a resilient health system [54]. A resilient system requires preparedness, responses, and recovery plans, including contingency plans and emergency regulations [49]. Planning clarifies roles and responsibilities and facilitates understanding of system structure and functions. An appropriate plan will lead to creating collaborative networks [41].

#### Monitoring

Monitoring of the outcomes health system and intermediate activities is an essential tool to reach health system resilience [14, 45, 50]. Monitoring uncontrolled or unwanted consequences of small changes is a necessity for anticipation [58, 61]. Also, early detection of any change signals through leading and lagging indicators will improve awareness and lead to an effective response [25, 62].

#### Information and communication systems

Information and communication systems are also vital tools for a resilient health system [20, 27, 28, 41, 49, 54]. The resilient health system provides communication links between network members and supports information and communication system infrastructures [54, 59] Also, risk communication protocols and feedback loops are two main functions of information and communication systems [10]. This tool will facilitate awareness, improve learning, collaboration and coordination, response, and preparedness against chronic challenges and shocks [7].

The effectiveness of information and communication systems depends on the availability of required data, quality of data, and essential infrastructures to transfer on-time information [7, 52]. Also, owning multiple information sources (Source resilience) will improve the reliability of information and communication systems [36, 37].

#### Learning

Learning from positive and negative experiences, success or failures, in extreme events or day-to-day activities, has a pivotal role in resiliency [14, 25, 27, 45, 50, 58, 59, 62, 65, 66]. Indeed, a resilient health system focuses on how to learn from events [52]. Effective learning provides appropriate analytical indicators to assist anticipation and monitoring [25]. In addition, learning will improve system responses and requires evaluation and feedback mechanisms [41]. Developing individuals and the entire system knowledge and skills to deal with the adverse condition through learning mechanisms, including training programs, practice, and experience, will lead to system resilience improvement [20, 33, 49, 66].

#### Institutionalization

Several researchers specified the importance of capacity building and providing necessary institutional software and hardware to respond, cope with and adapt to crisis conditions [48]. For example, Blanch et al. argued that a resilient system could create organizations that are socially accepted and contextually compatible and called it “legitimacy” which can be achieved through community engagement [10]. Also, Albanese et al. introduced institutional capacity building as one of the necessary infrastructures for building a resilient hospital and achieving minimum standards [33, 69].

### Health system resilience main strategies

#### Absorptive strategies

Absorptive strategies, also known as persistence or situated resilience strategies, protect the system against shocks and the impact of hazards, which are usually small-scale shocks or events [32, 62, 70]. These strategies return the system to its original state or reduce the severity and implications of the crisis on the system without making any particular change in structure, using available resources and capacities (skills, knowledge, tools, and data) [10, 30, 32, 44, 62, 70]. Such strategies are generally used in the response phase [62].

#### Adaptive strategies

Adaptive strategies, named structural resilience, can lead to a limited number of gradual adjustments in the structure or process of the system [32, 44, 62, 70]. These strategies promote service delivery at the same level before the crisis and maintain core system activities using less or different resources [10]. Adaptive strategies will apply for more intensive challenges which absorptive strategies can’t deal with [32].

#### Transformative strategies

Transformative strategies or systemic resilience create long-term and significant changes in the system structure and functions in response to massive environmental changes or challenges [10, 32, 44, 52, 62, 70].

### WHO health system six building blocks

#### Leadership and governance

Leadership can cause influence and be affected by resilient system attributes, strategies, and tools simultaneously. A resilient health system creates a transparent and flexible crisis leadership and governance structure [54, 71]. It chooses the leadership style according to the context [71]. The leadership and governance affect coordination and collaboration by identifying and employing various actors (for example between the private and public sectors in the health system). Also, it makes capacity for anticipation, planning, and institutionalization possible [41, 48]. The leadership and governance should focus on transparency, responsiveness, equity, and control and monitoring of other system components (service delivery, financing, human resources, etc) [41, 71].

#### Human resources

To achieve health system resiliency, we need a resilient workforce as well [41, 45]. The resilient health system workforce has flexibility, including redundancy, adequate health workforce, additional workforce, and health workforce with multiple skills [7, 41, 54]. In addition, they receive the necessary training to deal with various crises (learning tools), for example, communication and collaboration, as the basic skills to deal with the crisis [20, 49, 54].

#### Financing

Crisis financial management [49], sufficient financial capacity to deal with the crisis [20, 48], using diverse and sustainable financial resources (flexibility) [54], timely access to financial resources [54], effective allocation, and using current resources [7], and planning to distribute resources during the crisis [20], are essential tips for creating a resilient health system.

#### Information system

The possibility of timely knowledge and information exchange and the existence of quality monitoring systems will lead to improved policymaking and resiliency of the health system through improving preparedness, response, and accelerating adaptation to various shocks and chronic challenges [7, 33, 52]. A resilient health system has “source resiliency” which is the existence of multiple information sources that can improve policymakers’ understanding of the system status in the time of crisis [36, 37]. The information system is a prerequisite for monitoring and learning through all levels (including the workforce, patients, families, and healthcare providers) [62].

Also, preservation, maintenance, and safety assurance of information and communication systems are necessary for resiliency in the health system [49]. A robust information system will improve collaboration and coordination, such as creating client-based information systems that can improve the relationships between the health system and its client [10]. In summary, the resiliency of the health system depends on the resiliency of its networking capacity to receive accurate and timely information [27].

#### Service delivery

Reducing services or patient discharges are usually the first reactions of health systems against the crisis [27]. However, the resilient health system sustains a basic level of routine health services and provides additional services for the community [7, 49, 54]. Focus on preventive services before the crisis prepares the health system against shocks, changes, and challenges [7]. In this regard, establishing governance structures, such as safety committees or infection control committees were recommended [49].

#### Drug and equipment

Shortages of high-quality drugs and medical equipment are a common problem during the crisis [49]. Using network capacity or collaboration and coordination attributes will improve the efficiency and effectiveness of procurements [27].

## Discussion

General guidance on analysing and implementing health system resilience could be useful to decide how to practice in different contextual situations [7]. However, there is a need to promote resiliency from descriptive or subjective approaches toward an integrated theoretical approach. Thus, this study aimed to achieve a comprehensive model to analyse and guide achieving health system resiliency [11]. For this purpose, a critical interpretive synthesis was applied.

We include studies from various disciplines to strengthen health system resilience concept. Most of the studies focused on similar concepts in defining a resilient system. The review of published models, frameworks or theories, showed that each focused on one or two detected dimensions of operationalization of resiliency. For example, Kruke et al. [9] Hollnagel et al. [8] and Bruneau et al. [72], focused on resilient system attributes, also Blanche et al. [10] and Barasa et al. [6] focused on resilient system strategies and attributes, and Rogers focused on phases of reaching resiliency [73].

Therefore, we concluded that a combination of different dimensions of resilience models, frameworks, and theories are necessary for its analysis and operationalization. Several studies tried to introduce a comprehensive model. In this regard, Wiig et al., in their study, referred to four dimensions to define and research the resilience concept including the purpose of resiliency, activators, and triggers of resilience, system components, and finally processes, activities and mechanisms to enable system resiliency [53]. Also, Thomas et al. defined resilience by three dimensions of preparation, management (absorptive, adaptive, and transformative strategies), and learning (recovery).

**Fig. 2:**
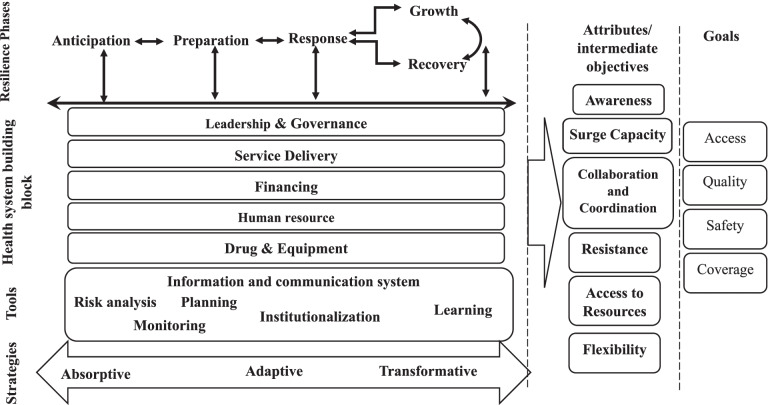
The Health System Resiliency Analysis Framework

The results of this study indicated five main themes to explain and analyze health system resilience (Fig. 2). The relationship and amount of investment in different dimensions determine based on the type of stressors and underlying conditions [44].

Policymakers and researchers can analyze and formalize their resilient system roadmap considering these dimensions. First, Resilience phases are introduced in order. However, as the system moves toward resilience, these phases will proceed continuously and in relation to each other. Second, considering the importance of the resilience concept as a dynamic health system objective to achieve health system goals [53, 74], resilient system attributes, also named “intermediate objectives” and health system goals are introduced. Third, to determine the boundaries of analysis [20, 25, 44, 45, 48, 71], the WHO health system’s six building blocks are considered as a necessary dimension for the analysis. Finally, the resilient system enablers’ identification is the prerequisite to deeper understanding and analyzing system resilience [53, 75]. Therefore, in our model, these enablers are introduced as resilience system tools that will be used as an absorptive, adaptive, or transformative strategy.

## Conclusion

This meta-framework provides the opportunity to analyze and identify the strengths and weaknesses of the system in achieving resilience in different contexts. For this purpose, the system’s performance will be examined against the crisis in each phase using appropriate indicators. This analysis will be conducted separately in each system building block. Also, applying different resilience tools such as risk analysis, monitoring, information and communication systems, learning, and institutionalization will be assessed. Consequently, the health system will be judged in terms of achieving resilient system attributes and then the health system goals. Finally, related policies in each dimension can be suggested.

## Supplementary Information

**Additional file 1.** PRISMA 2020 Checklist.

## Data Availability

All data generated or analysed during this study are included in this published article [and its supplementary information files].

## Abbreviations

WHO: World Health Organization
PRISMA: Preferred Reporting Items for Systematic Reviews and Meta-Analyses
COVID-19: Corona Virus Disease of 2019
